# Comparison of Nasopharyngeal and Saliva Swab Nucleic Acid Amplification and Rapid Antigen Testing To Detect Omicron SARS-CoV-2 Variant of Concern: a Prospective Clinical Trial (OMICRON)

**DOI:** 10.1128/spectrum.03923-22

**Published:** 2022-11-08

**Authors:** Antonios Kritikos, Giorgia Caruana, Catherine Lazor-Blanchet, Michael Currat, Jean-Daniel Chiche, Peter Vollenweider, Pierre-Alexandre Bart, Onya Opota, Gilbert Greub

**Affiliations:** a Institute of Microbiology, Lausanne University Hospital and University of Lausanne, Lausanne, Switzerland; b Occupational Health Service, Lausanne University Hospital, Lausanne, Switzerland; c Intensive Care Unit, Lausanne University Hospital and University of Lausanne, Lausanne, Switzerland; d Service of Internal Medicine, Lausanne University Hospital and University of Lausanne, Lausanne, Switzerland; University of Cincinnati

**Keywords:** SARS-CoV-2 diagnosis, Omicron variant, RT-PCR, rapid antigen testing, nasopharyngeal swab, saliva swab

## Abstract

In November 2021, the World Health Organization declared the Omicron variant (B.1.1.519) a variant of concern. Since then, worries have been expressed regarding the ability of usual diagnostic tests to detect the Omicron variant. In addition, some recently published data suggested that the salivary reverse transcription (RT)-PCR might perform better than the current gold standard, nasopharyngeal (NP) RT-PCR. In this study, we aimed to compare the sensitivities of nasopharyngeal and saliva RT-PCR and assess the diagnostic performances of rapid antigen testing (RAT) in nasopharyngeal and saliva samples. We conducted a prospective clinical study among symptomatic health care professionals consulting the occupational health service of our hospital for severe acute respiratory syndrome coronavirus 2 (SARS-CoV-2) screening and hospitalized patients in internal medicine/intensive care wards screened for SARS-CoV-2 with COVID-19-compatible symptoms. A composite outcome considering NP PCR and/or saliva PCR was used as a reference standard to define COVID-19 cases. A total of 475 paired NP/saliva specimens have been collected with a positivity rate of 40% (*n* = 192). NP and salivary RT-PCR exhibited sensitivities of 98% (95% CI, 94 to 99%) and 87% (95% CI, 81 to 91%), respectively, for outpatients (*n* = 453) and 94% (95% CI, 72 to 99%) and 69% (95% CI, 44 to 86%), respectively, for hospitalized patients (*n* = 22). Nasopharyngeal rapid antigen testing exhibited much lower diagnostic performances (sensitivity of 66% and 31% for outpatients and inpatients, respectively), while saliva RAT showed a sensitivity of less than 5% in both groups. Nasopharyngeal RT-PCR testing remains the gold standard for SARS-CoV-2 Omicron variant screening. Salivary RT-PCR can be used as an alternative in case of contraindication to perform NP sampling. The use of RAT should be limited to settings where access to molecular diagnostic methods is lacking.

**IMPORTANCE** The Omicron variant of concern spread rapidly since it was first reported in November 2021 and currently accounts for the vast majority of new infections worldwide. Recent reports suggest that saliva sampling might outweigh nasopharyngeal sampling for the diagnosis of the Omicron variant. Nevertheless, data investigating the best diagnostic strategy specifically for the Omicron variant of concern remain scarce. This study fills this gap in current knowledge and elucidates the question of which strategy to use in which patient. It provides a new basis for further improving COVID-19 screening programs and managing patients suspected to have COVID-19.

## INTRODUCTION

The Omicron variant of concern (B.1.1.529, BA lineage) was first reported from Botswana on November 11, 2021, and soon thereafter from South Africa (1). As of late December 2021, Omicron accounted for the majority of new infections worldwide (2). Asthenia, cough, fever, headache, runny nose, and sore throat are the most frequently reported symptoms, while loss of smell and taste are reported only sporadically (3–6). This variant has been associated with increased transmissibility and appears to have a greater replication rate than previous circulating variants (7). The omicron variant has over 30 mutations in the spike protein and in particular in the receptor-binding domain affecting its binding affinity and conferring higher antibody escape (8–10). Worries have initially been expressed regarding the ability of diagnostic tests to detect the Omicron variant. Analyses performed so far, however, suggest that the gold-standard PCR tests remain highly effective in Omicron detection (9, 11–13). This is also valid for rapid antigen testing (RAT) (11). The optimal diagnostic strategy to detect Omicron variant remains yet unknown, and recent publications report conflicting results (11, 12). While nasopharyngeal (NP) swabs have so far been considered the gold standard for severe acute respiratory syndrome coronavirus 2 (SARS-CoV-2) diagnosis (14–16), there is evidence in the literature suggesting that saliva sampling might outweigh NP swab for the diagnosis of the Omicron variant (12, 17, 18).

In order to determine the best diagnostic strategy for SARS-CoV-2 Omicron variant diagnosis, we conducted a prospective observational study among symptomatic individuals in our institution. Our aims were to compare sensitivity of NP versus saliva reverse transcription (RT)-PCR and to evaluate the diagnostic performances of rapid antigen testing in NP and saliva samples.

## RESULTS

### Patient characteristics.

All health care employees with COVID-19-compatible symptoms that presented during the study period to our hospital’s screening center were screened for eligibility criteria. A total of 453 paired NP and saliva swabs were taken with a positivity rate of 39% (*n* = 176). A total of 22 inpatients with new onset COVID-19-compatible symptoms were also screened. The prevalence of SARS-CoV-2 infection among hospitalized patients was 73% (*n* = 16). Baseline demographics and clinical characteristics are shown on Table 1. The patients were predominantly female (*N* = 322, 68%) with a median age of 38 years old (interquartile range [IQR], 31 to 49 years old). The median duration of symptoms before testing was 1 day (IQR, 1 to 2 days). The most common symptoms on presentation were sore throat (*N* = 294, 62%), nasal congestion (*N* = 268, 57%), headache (*N* = 241, 51%), fatigue (*N* = 225, 48%), and cough (*N* = 205, 43%).

**TABLE 1:** Demographics and clinical characteristics of participants

Characteristics	SARS-CoV-2 negative (*N* = 283)^*a*^	SARS-CoV-2 positive (*N* = 192)^*a*^	*P* value
Patient group			
Hospitalized patients	6 (2.1%)	16 (8.3%)	
Health care workers	277 (98%)	176 (92%)	
Age	36 (30, 48)	39 (32, 52)	0.11
Gender			0.57
Female	189 (67%)	133 (69%)	
Male	94 (33%)	59 (31%)	
Food or drink consumption before testing	72 (28%)	35 (21%)	0.12
Symptoms’ duration	1 (1, 2)	1 (1, 2)	0.43
Sore throat	166 (59%)	128 (68%)	**0.046**
Cough	92 (33%)	113 (60%)	**<0.001**
Nasal congestion	155 (55%)	113 (60%)	0.28
Fatigue	135 (48%)	90 (48%)	0.99
Muscle aches	83 (29%)	58 (31%)	0.75
Fever	27 (9.5%)	31 (16%)	**0.026**
Chest pain	9 (3.2%)	14 (7.4%)	**0.037**
Anosmia/ageusia	4 (1.4%)	5 (2.6%)	0.49
Headache	146 (52%)	95 (50%)	0.78
Gastrointestinal symptoms	48 (17%)	24 (13%)	0.21
Known contact with a positive case	80 (30%)	69 (40%)	**0.023**
No. of received vaccination doses			0.14
0	24 (8.5%)	25 (13%)	
1	18 (6.4%)	7 (3.7%)	
2	49 (17%)	40 (21%)	
3	192 (68%)	117 (62%)	
Oxygen saturation	98 (97, 98)	98 (97, 98)	**0.022**

a Median (interquartile range); n (%); Wilcoxon rank sum test; Pearson’s chi-square test. *P* values that are statistically significant (< 0.05) are highlighted in bold.

### Diagnostic performance of RT-PCR and rapid antigen testing.

A positive NP or salivary RT-PCR was considered the reference standard comparator for the definition of COVID-19-positive cases. Among health care workers, 453 paired NP/saliva samples were analyzed with RT-PCR, and 449 paired samples were analyzed with RAT. In four cases, RAT was not performed because the viral transport medium (VTM) was discarded before realization of the test. Among the performed RAT, two were tested beyond 72 h and were therefore excluded from the analysis.

**FIG 1:**
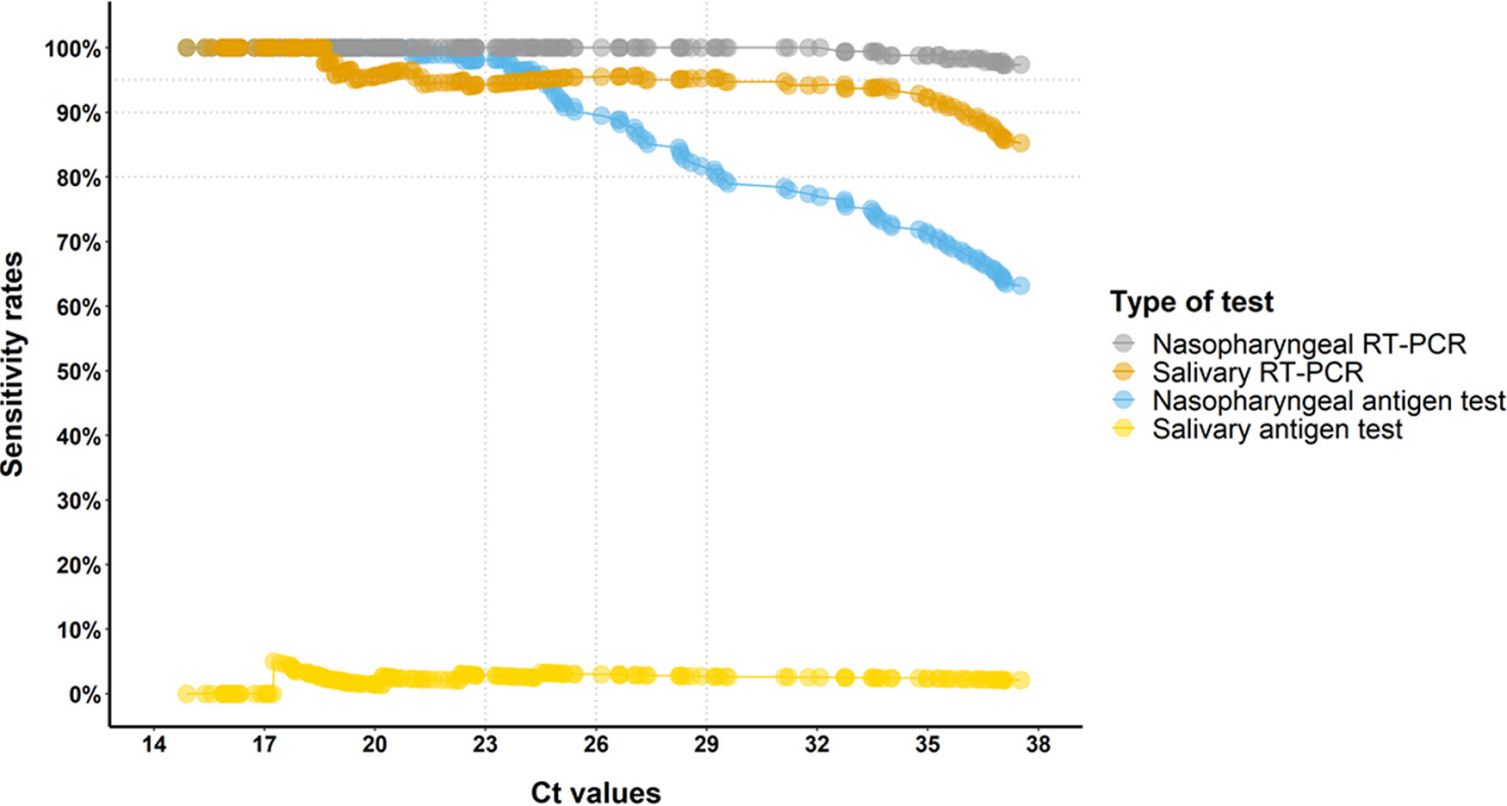
Sensitivity according to reverse transcription (RT)-PCR cycle threshold of health care workers. On the *x* axis, there is the number of cycle threshold (Ct); horizontal lines represent the Ct cutoffs considered for the rapid antigen testing (RAT) validation criteria according to the Swiss Society of Microbiology (SSM). Horizontal dotted lines represent the threshold of minimal sensitivity considered for RAT validation at different Ct cutoffs according to SSM criteria.

The diagnostic performance of RT-PCR and RAT for the diagnosis of SARS-CoV-2 infection in this population is shown on Table 2. Among health care workers, NP and salivary RT-PCR exhibited overall sensitivities of 98 and 87%, respectively. NP RAT exhibited much lower sensitivity (66%), while the specificity was at 100%. Salivary RAT showed a sensitivity of 2%. The sensitivity rates remained stable independently of cycle threshold (Ct) values for RT-PCR, while the sensitivity of NP RAT decreased significantly above 26 Ct (Fig. 1).

**TABLE 2:** Overall diagnostic performance of RT-PCR and rapid antigen test performed on nasopharyngeal or salivary samples^*a*^

Patient group	Diagnostic test	Prevalence (%)	Sensitivity [95% CI] (%)^*b*^	Specificity [95% CI] (%)	PPV [95% CI] (%)	NPV [95% CI] (%)
Health care workers (*n* = 453)	Nasopharyngeal RT-PCR	38	98 [94 to 99]	n.a.^*c*^	n.a.^*c*^	98 [96 to 99]
	Salivary RT-PCR	34	87 [81 to 91]	n.a.^*c*^	n.a.^*c*^	92 [89 to 95]
	Nasopharyngeal RAT	26	66 [59 to 73]	100 [98 to 100]	100 [97 to 100]	100 [78 to 86]
	Salivary RAT	1	2 [0.5 to 5]	99 [97 to 100]	60 [23 to 93]	61 [56 to 65]
Hospitalized patients (*n* = 22)	Nasopharyngeal RT-PCR	68	94 [72 to 99]	n.a.^*c*^	n.a.^*c*^	86 [49 to 99]
	Salivary RT-PCR	50	69 [44 to 86]	n.a.^*c*^	n.a.^*c*^	54 [28 to 79]
	Nasopharyngeal RAT	23	31 [14 to 56]	100 [61 to 100]	100 [57 to 100]	35 [17 to 59]
	Salivary RAT	4.5	6 [1 to 28]	100 [61 to 100]	100 [5 to 100]	29 [14 to 50]

*^a^*CI, confidence interval; PPV, positive predictive value; NP, nasopharyngeal; NPV, negative predictive value, n.a., not applicable; RAT, rapid antigen testing; RT, reverse transcription.

*^b^*Diagnostic performances were evaluated based on a composite outcome considering NP PCR and/or saliva PCR as a COVID-19 case.

*^c^*Calculation of specificity and PPV is not applicable, since a positive NP PCR and/or saliva PCR are part of the definition of the composite outcome.

Among inpatients, 22 paired NP/saliva samples were evaluated with RT-PCR and RAT. The diagnostic performance of RT-PCR and RAT for the diagnosis of SARS-CoV-2 infection in this population is shown on Table 2. NP and salivary RT-PCR exhibited overall sensitivities of 94 and 69%, respectively. NP RAT exhibited much lower sensitivity (31%), while the specificity was at 100% (95% CI, 54 to 100). Salivary RAT showed a sensitivity of 6%.

### Predictive agreement of SARS-CoV-2 detection in nasopharyngeal and saliva swabs.

Paired NP and saliva swab results of RT-PCR from health care workers are shown in Table 3. An analysis of the agreement between the two specimens (NP versus saliva) revealed very good agreement, with a κ coefficient of 0.87 and an overall proportion of agreement of 94% (95% CI, 91 to 96) (Table 3). When sensitivities were compared between both sample types using a McNemar test, we documented a higher sensitivity for NP swab (*P* < 0.001). Discordant results were observed among 27 cases, with 23 saliva and 4 NP samples showing negative results, while the other specimen tested positive (Table 3). All four patients with negative NP and positive saliva sample were fully vaccinated and reported sore throat and fatigue as the main symptoms. Three of these four patients consulted within 36 h of their symptoms’ appearance.

**TABLE 3:** Agreement matrix of matched NP and saliva swabs from health care workers^*a*^

SARS-CoV-2 RT-PCR	NP swab		Total
	Positive	Negative	
Saliva swab		
Positive	149	4	153
Negative	23	277	300
Total	172	281	453

*^a^*Only RT-PCR results from health care workers are depicted in this table. Positive agreement (95% CI) was 87% (81 to 91%), negative agreement was 98% (96 to 99%), and overall agreement was 94% (91 to 96%). *P* values were determined with McNemar tests.

### SARS-CoV-2 viral loads in nasopharyngeal and saliva swabs.

**FIG 2:**
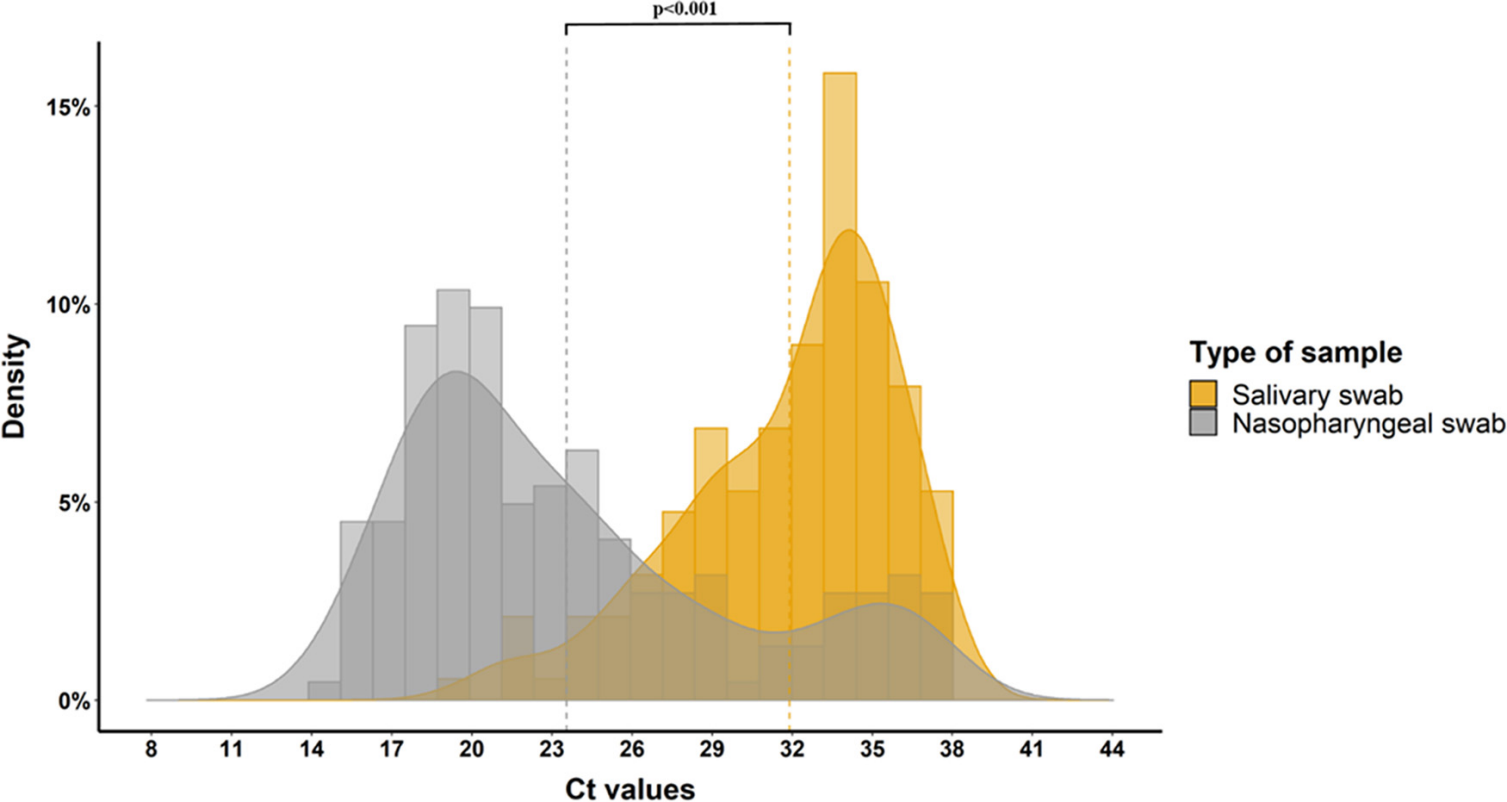
Histograms and density plots of observations’ distribution according to Ct values. The yellow (Ct 23.2) and gray (Ct 32) vertical dotted lines represent median Ct values for the nasopharyngeal (NP) and salivary swab groups, respectively: a statistical significant difference was found when comparing the medians (Wilcoxon signed-rank test *P* < 0.001).

**FIG 3:**
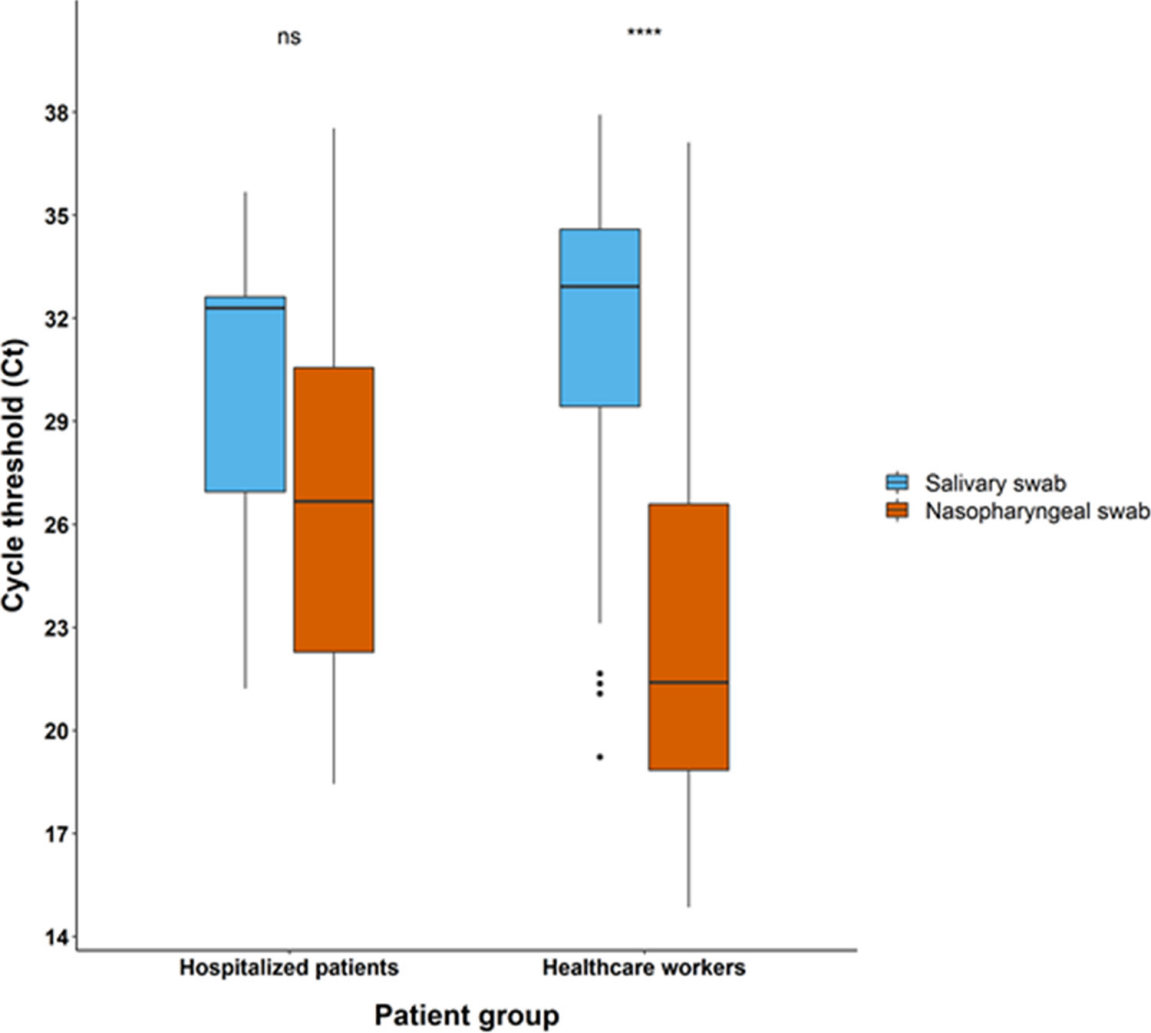
Box plots of Ct values according to sampling site for the two groups of patients. ns, not significant; ****, *P* < 0.0001.

**FIG 4:**
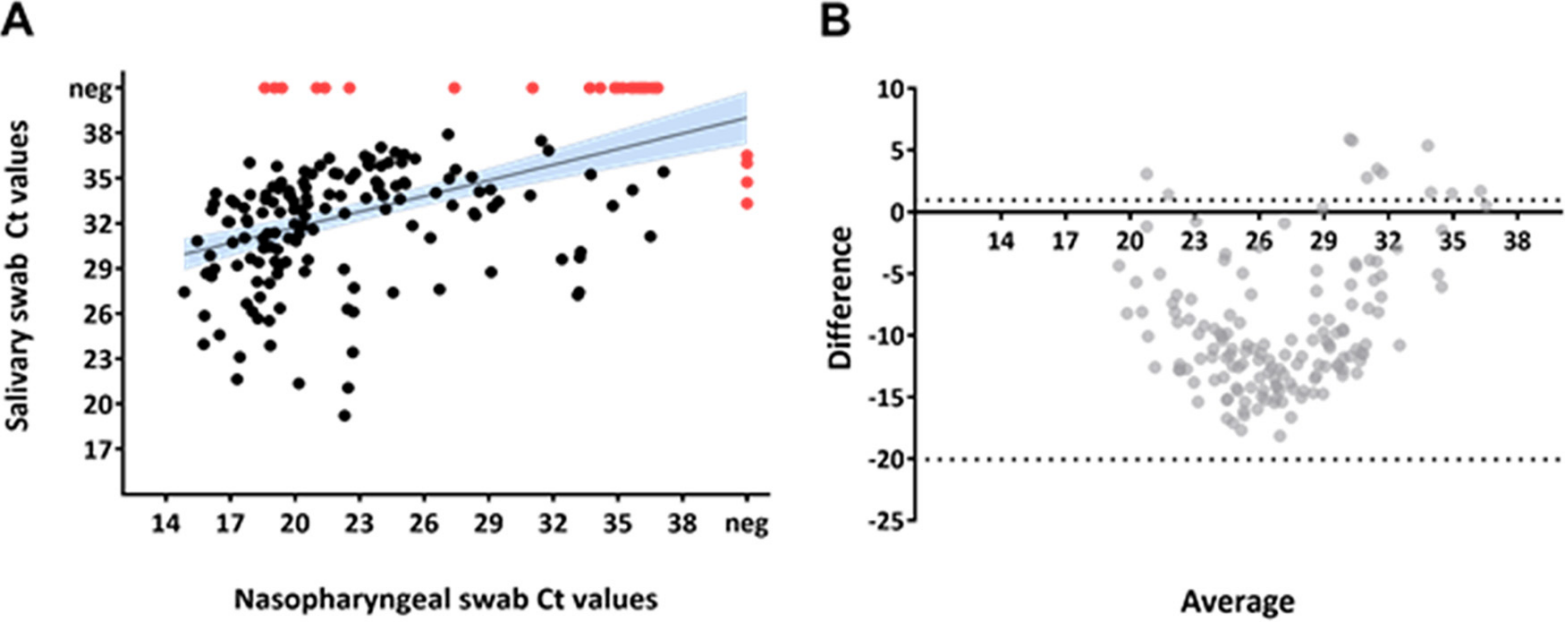
Correlation of SARS-CoV-2 levels in NP and saliva swabs from health care workers. (A) Scatter plot of paired NP and saliva swabs Ct values. Black line with blue shaded areas show linear trend with 95% confidence intervals. Red dots show samples that yielded negative results with one or the other method. (Pearson *r* value = 0.48, 95% CI 0.36, *P* < 0.001). (B) Bland-Altmann plot Ct values of NP and saliva swabs of all positive pairs. The plot shows the difference between the two measurements on the *y* axis and the average of the two measurements on the *x* axis. Black dashed lines equal 95% limits of agreement. (Bias = −9.3, SD of bias = 5.9, 95% limits of agreement from −20.9 to 2.3).

**FIG 5:**
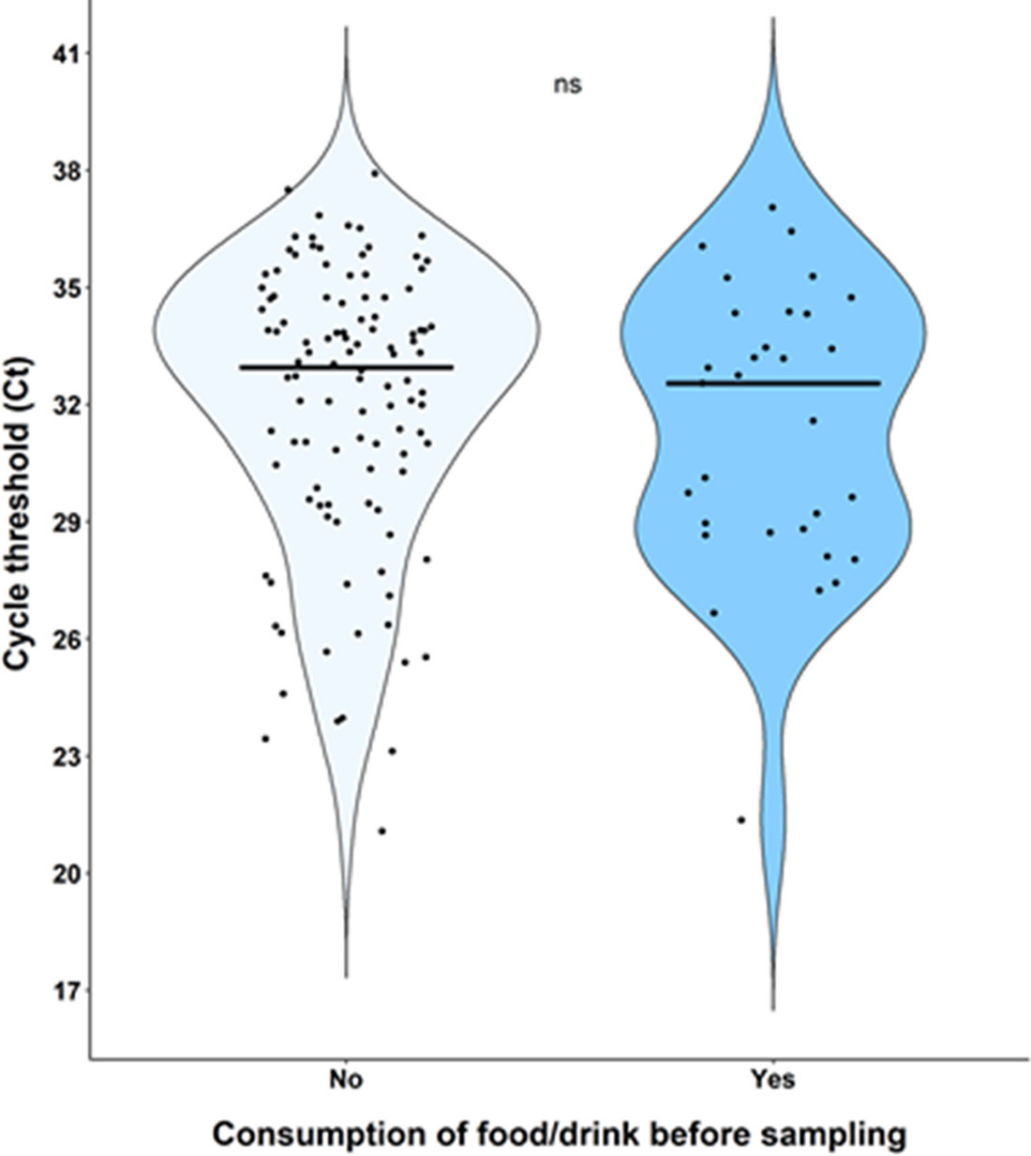
Impact of food/drink consumption before saliva sampling on Ct values. ns, not significant.

The viral load in NP swabs was higher than that detected in salivary specimens (Fig. 2) for both health care workers and hospitalized patients (Fig. 3). Among health care workers, the correlation analysis of sample pairs showed that NP and saliva results were in good agreement (Pearson r value = 0.48, *P* value < 0.001) (Fig. 4A). Notably though, the Ct values in saliva were on average 9.3 times higher than the corresponding Ct in NP swabs (Fig. 4B). To investigate whether this difference among NP and saliva swabs viral loads could be explained by any consumption of food/drink or smoking before saliva sampling, we stratified saliva swabs according to the above-mentioned conditions. Food or drink consumption or smoking did not influence the viral load in saliva swabs (Fig. 5).

### Correlation of SARS-CoV-2 viral loads with symptoms or immunization status.

**FIG 6:**
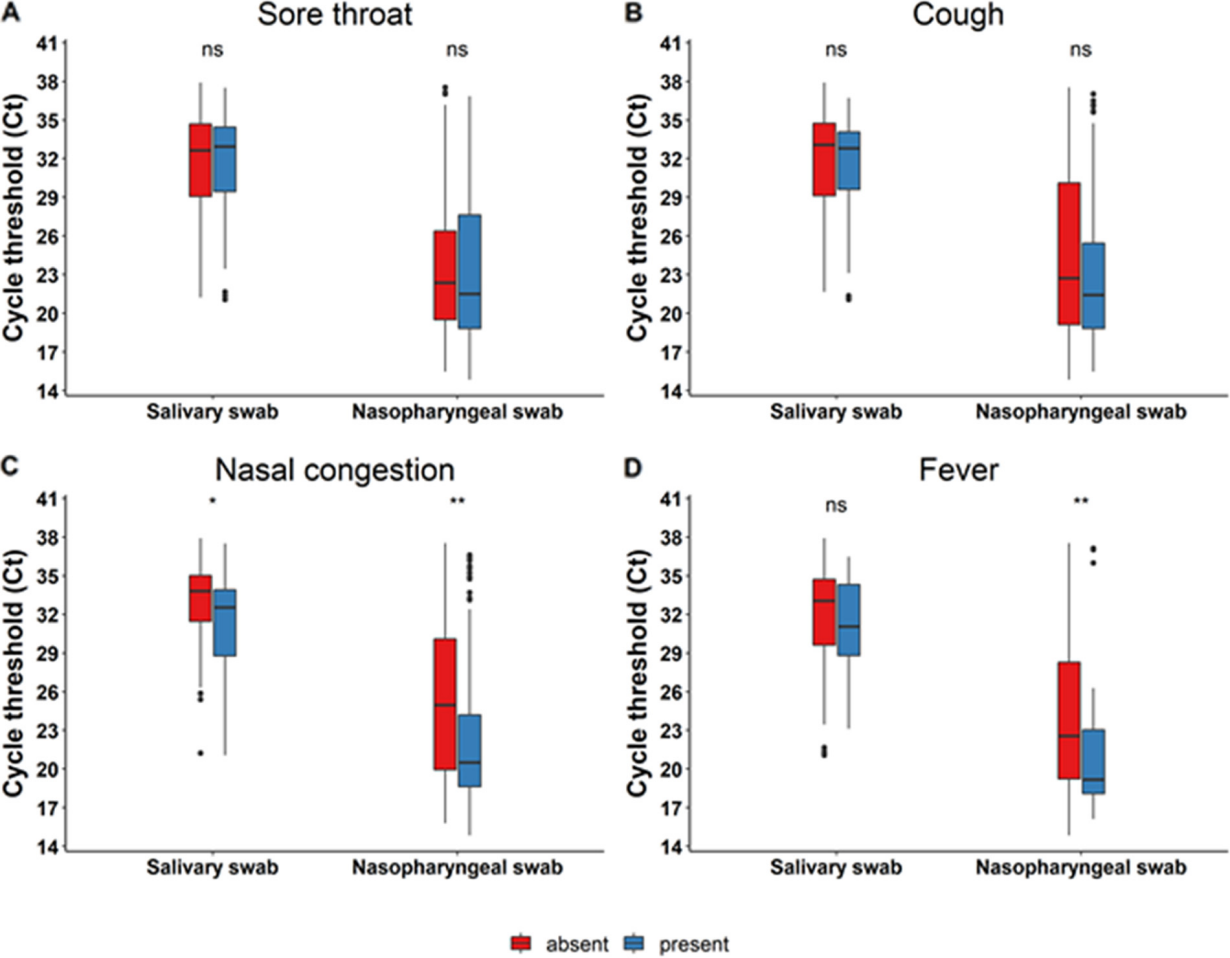
Correlation of Ct values with symptoms. Panels (A), (B), (C), and (D) show correlation of Ct values with sore throat, cough, nasal congestion and fever respectively. “Absent” or “present” in the legend refer to the absence or presence of the symptom of concern respectively. ns, not significant; *, *P* < 0.05; **, *P* < 0.01.

Our study recorded the patients’ symptoms and immunization status. We observed a statistically significant trend toward higher viral loads (i.e., lower Ct values) in symptomatic patients with nasal congestion (Fig. 6C) with both NP and salivary samples. Febrile patients also had significantly higher viral loads (i.e., lower Ct values) in nasopharyngeal swabs (Fig. 6D). Immunization status did not have an impact on viral load documented both in NP and saliva swabs.

## DISCUSSION

Our study, performed mainly on a population of COVID-19-symptomatic health care workers, showed a good agreement between NP and salivary RT-PCR. SARS-CoV-2 viral load in the NP tract was significantly higher than salivary viral load (22.2 versus 32 Ct, *P* < 0.001), with food/drink consumption or smoking prior to sampling having no influence. Analysis of paired NP and saliva samples from a small group of hospitalized patients showed a similar trend in viral load as of outpatients, though without reaching statistical significance. Moreover, viral loads were higher when assessing patients with nasal congestion and fever compared to patients without those symptoms. Only 5 (1%) patients resulted positive on salivary swabs and negative on NP samples (4 outpatients and 1 inpatient), a result that is concordant with usual false-negative rates of NP PCR. Some recent data suggest that viral loads could rise faster in saliva than in NP samples (17), potentially explaining part of the discordant results especially early in the course of the disease. In our study, 3 out of 5 of the above-mentioned discordant results were patients presenting at screening within 24 h since symptom onset. On the other hand, NP sampling is more laborious to perform and demands a better expertise than saliva sampling. This could also potentially explain part of the discordant results in favor of saliva sampling as demonstrated in some studies. Nevertheless, in our study, all NP and saliva health care workers’ samples were performed by an experienced staff member dedicated to performing the screening sampling since the beginning of the pandemic and therefore making this last hypothesis less likely.

Our results, while corroborating NP swab as the gold-standard sample for SARS-CoV-2 Omicron screening (14–16), are in contrast with recent data showing a benefit of salivary samples for Omicron variant (12, 17, 18). Importantly, in those other studies, either the number of patients with the Omicron variant was not disclosed (12) or the sample size was too small to allow any statistical interpretation (17).

Based on our findings, RT-PCR on a salivary sample can represent a reliable alternative to RT-PCR on NP samples, at least for routine analysis for SARS-CoV-2 Omicron screening, especially for patients with any contraindications to NP sampling. For outpatient screening, salivary RT-PCR represents a fair approach with 87% sensitivity. For routine screening at the beginning of hospitalization, the gold-standard nasopharyngeal swab should still be preferred in the absence of contraindication to avoid nosocomial spread caused by a missed case using this approach. For the follow-up of already diagnosed cases, however, saliva RT-PCR could be a fair, noninvasive alternative to NP RT-PCR. An alternative approach that is considered by some centers is to pool salivary and NP swabs to compensate for the 1% false-negative NP RT-PCR. Nevertheless, a formal evaluation for this complementary strategy should be carried out, especially in order to make sure that RNases in the saliva do not affect the NP viral load.

Our results also showed a lower sensitivity of RAT compared to RT-PCR: 67% versus 98%, respectively, among health care workers with 1 to 2 days of COVID-19-like symptoms and 31% versus 94% among hospitalized patients with a median duration of symptoms of 10 days. In both populations, salivary RAT was considered insufficient for SARS-CoV-2 screening, showing a sensitivity of less than 5%. These findings reflect previous data on SARS-CoV-2 non-Omicron variants showing the lower sensitivity of RAT (14, 19, 20) compared to NP RT-PCR and do not demonstrate a lower sensitivity of RAT for Omicron compared to other variants.

There are a few limitations to our study. First, we were unable to assign a specific variant to 37 positive samples (either due to low viral load [*n* = 31] or genome sequencing failure [*n* = 6]). Those samples were included in the analysis and could therefore have an impact on our results. Nevertheless, nationwide epidemiological surveillance data during the study period reported that Omicron variant of concern exceeded 99% of all circulating variants.

Second, it is worth mentioning that there is no consistent best practice for saliva sampling in the framework of COVID-19 infection, and many different methods have been used to do this collection. The different studies that evaluated saliva as a diagnostic method are difficult to compare because they have used various collection techniques. In this study, we collected saliva samples based on the recommendations of the European Centre for Disease Prevention and Control (ECDC) (21) and previously published data (14, 22, 23). We cannot exclude that another saliva collection technique might have performed differently in this context, and further prospective comparative studies are needed to elucidate this question. Moreover, for hospitalized patients, we accepted a maximum delay of 6 h for the collection of the two samples (NP and saliva swab). While this seems a reasonable delay for the realization of the two samples, it might have impacted the yield of saliva samples. Hospitalized patients also had a longer duration of symptoms in comparison to outpatients (median of 10 days versus 1 day), which explains the differences in observed viral loads (14, 19).

The application of RAT on saliva is not currently clinically validated and was performed off-label for this study (not according to the manufacturers’ instructions for use). We cannot exclude antigen inhibition or degradation in saliva samples, which could eventually explain the very low diagnostic performance of RAT on saliva.

In conclusion, it is clear that in a context of SARS-CoV-2 Omicron predominance, NP RT-PCR testing remains essential for screening for SARS-CoV-2 infection, as well as to monitor the population for the arrival of new variants. A salivary sample for RT-PCR can be used as a substitute (or complementary) to NP swab in specific contexts. RAT has been a useful resource for outpatient testing in the context of massive screening during periods of high prevalence of disease and rapid PCR reagent shortage, in order to relieve laboratories from the high workload and still guarantee a first screening (further confirmed by molecular methods). Nevertheless, nowadays, due to their lower sensitivity and the constant need of new variant monitoring, their use should be limited to settings lacking access to diagnostic laboratories with molecular methods, and their results, especially if negative, should be corroborated by a RT-PCR test.

## MATERIALS AND METHODS

### Study population.

We conducted a prospective clinical study among health care professionals consulting the occupational health service and hospitalized patients in internal medicine or intensive care wards screened for SARS-CoV-2 infection. All participants were symptomatic with COVID-19-compatible symptoms. The study took place at Lausanne University Hospital, a 1,500-bed tertiary care center in Switzerland, between February 4, 2022, and March 31, 2022. The demographics and clinical data including each patient’s clinical presentation and duration of symptoms, immunization status, and underlying conditions were collected in a specific case report form.

### Inclusion and exclusion criteria.

Subjects meeting the following criteria were eligible for inclusion in the study: (i) age ≥18 years and (ii) COVID-19-compatible symptoms (sore throat, nasal congestion, headache, fatigue, cough, muscle aches, etc.). Subjects unable to provide written informed consent or with contraindications to perform NP or salivary swab were excluded from the study.

### Sample collection and diagnostic tests.

After having given their informed consent, all study participants underwent a NP (24) and a salivary swab (14, 22, 23) by a health care professional according to ECDC guidelines for clinical specimen collection (21) and previously published data (14, 22, 23). Regarding saliva sampling, briefly, the caregiver collected a significant amount of saliva by swabbing the upper and lower gingival space, the cheeks on both sides, and under the tongue to end with the hard and soft palate, and this for at least 20 s (supplemental material). All swabs were collected using a flocked swab and transported within 3 mL universal viral transport medium (UTM Copan Diagnostics).

Health care professionals who presented to our hospital’s occupational medicine service for SARS-CoV-2 screening underwent simultaneous NP and salivary sampling. For hospitalized patients, we implemented an automated alert system that notified the investigators each time a patient hospitalized in one of the participating wards was screened for SARS-CoV-2. Eligible patients were subsequently proposed to undergo an additional salivary swab with a maximum delay of 6 h between the realization of the two samples.

Paired NP and saliva samples were analyzed by RT-PCR (Cobas 6800, Roche Switzerland) targeting the E and RdRp genes (25) and by RAT detecting the nucleocapsid protein (N) antigen (Standard Q COVID-19 rapid antigen test [Roche Switzerland]). Rapid antigen testing was performed within 72 h from sampling (maximum accepted delay to account for samples received at the end of the week that were not treated during the weekend) on specimens previously soaked in universal VTM (UTM Copan Diagnostics) (“wet approach”) according to the manufacturer’s instructions (26). Previously published data showed comparable diagnostic performances of rapid antigen testing performed using the wet approach compared to rapid antigen testing performed directly from NP swabs (called the “dry approach”) (14).

During the study period, public health data from nationwide epidemiological surveillance reports showed that the Omicron variant of concern (B.1.1.529) represented more than 99% of circulating variants (27). In our study, diagnostic screening with RT-PCR confirmed the S-gene dropout on 90% (138 of 154) of screened subjects (due to a 69–70del mutation) (8), likely belonging to Omicron BA.1 lineage according to the local genomic surveillance (data not published). The remaining 10% (*n* = 16) of subjects screened and not presenting the S dropout underwent genomic analysis and were classified as Omicron BA.2 variant. Of note, 37 positive samples were either not screened due to low viral load (*n* = 31) or had S-gene dropout screening and/or genome sequencing failure (*n* = 6). DNA was extracted using the automated system MagNaPure 96 (Roche), and the screening of the Omicron variant was achieved with the VirSNiP mutation assays DEL69/70 (TIBMOLBIOL) using a QuantStudio 7 instrument (Thermo Fisher).

### Statistical analysis.

Descriptive statistics are presented as the number and percentage for categorical variables and mean ± standard deviation (SD) or median (interquartile range [IQR]) for continuous variables. Chi-square or Fisher’s exact tests were used for categorical variables and Wilcoxon matched-pairs rank test for continuous variables, where appropriate. Sensitivity, specificity, positive and negative predictive values, and 95% confidence interval (CI) were calculated to assess diagnostic performances using a positive NP or saliva RT-PCR as a reference standard. The overall agreement between NP and saliva RT-PCR was assessed using the κ Cohen’s coefficient, and the sensitivity of both methods was compared using McNemar’s test. All statistical analyses were performed using the R software v4.1.1 (R Foundation for Statistical Computing; www.r-project.org) (28) or GraphPad Prism version 9.1.0 for Windows (GraphPad Software, San Diego, CA, USA).

### Ethics.

This project was conducted in accordance with the Declaration of Helsinki, the principles of Good Clinical Practice and the Swiss Human Research Act (HRO). The project received approval from the Ethics Committee of Canton Vaud, Switzerland (2022-00173).

### Data availability.

The data supporting results will be available upon request for the peer-review process.
